# Sequence Capture and Phylogenetic Utility of Genomic Ultraconserved Elements Obtained from Pinned Insect Specimens

**DOI:** 10.1371/journal.pone.0161531

**Published:** 2016-08-24

**Authors:** Bonnie B. Blaimer, Michael W. Lloyd, Wilson X. Guillory, Seán G. Brady

**Affiliations:** 1 Department of Entomology, National Museum of Natural History, Smithsonian Institution, Washington, District of Columbia, United States of America; 2 Department of Biological Sciences, University of Arkansas, Fayetteville, Arkansas, United States of America; University of California Berkeley, UNITED STATES

## Abstract

Obtaining sequence data from historical museum specimens has been a growing research interest, invigorated by next-generation sequencing methods that allow inputs of highly degraded DNA. We applied a target enrichment and next-generation sequencing protocol to generate ultraconserved elements (UCEs) from 51 large carpenter bee specimens (genus *Xylocopa*), representing 25 species with specimen ages ranging from 2–121 years. We measured the correlation between specimen age and DNA yield (pre- and post-library preparation DNA concentration) and several UCE sequence capture statistics (raw read count, UCE reads on target, UCE mean contig length and UCE locus count) with linear regression models. We performed piecewise regression to test for specific breakpoints in the relationship of specimen age and DNA yield and sequence capture variables. Additionally, we compared UCE data from newer and older specimens of the same species and reconstructed their phylogeny in order to confirm the validity of our data. We recovered 6–972 UCE loci from samples with pre-library DNA concentrations ranging from 0.06–9.8 ng/μL. All investigated DNA yield and sequence capture variables were significantly but only moderately negatively correlated with specimen age. Specimens of age 20 years or less had significantly higher pre- and post-library concentrations, UCE contig lengths, and locus counts compared to specimens older than 20 years. We found breakpoints in our data indicating a decrease of the initial detrimental effect of specimen age on pre- and post-library DNA concentration and UCE contig length starting around 21–39 years after preservation. Our phylogenetic results confirmed the integrity of our data, giving preliminary insights into relationships within *Xylocopa*. We consider the effect of additional factors not measured in this study on our age-related sequence capture results, such as DNA fragmentation and preservation method, and discuss the promise of the UCE approach for large-scale projects in insect phylogenomics using museum specimens.

## Introduction

Traditionally, a major goal of natural history museums has been to preserve specimens for morphological and taxonomic studies. With the advent of Sanger sequencing and the beginning of the molecular revolution in systematics and phylogenetics, the role of natural history collections in the 21th century has extended well beyond preserving morphological taxonomy to becoming a resource for genomic tissue samples [1,2,3]. However, the degradation of DNA in historically collected museum specimens can render them difficult or impossible to use in molecular phylogenetics [4,5]. Thus, most studies published to date have relied on recently collected specimens sampled specifically with the intent of preserving tissue for molecular genetics. New field collections can be an expensive endeavor when the focus is a rare or worldwide-distributed group of organisms, and, especially for diverse groups of insects and other arthropods, it may take many years to acquire the desired taxon sampling.

Interest in utilizing existing museum collections for molecular studies is on the rise, especially given the increasing availability of target capture and next-generation sequencing (NGS) techniques that capitalize on shorter, more degraded DNA fragments and thus circumvent some of the problems associated with Sanger sequencing of old material e.g., [6,7,8,9,10]. For example, sequence capture approaches have been applied to museum specimens >100 years of age in bats [11], birds [12], mammals [8,13,14], insects [10], and plants and fungi [6]. One issue, especially for small insects, is that degradation of DNA quality within a sample not only leads to fragmentation of DNA but also reduces DNA extraction yield [15]. Most molecular studies on arthropod museum specimens have so far focused mainly on sequencing mitochondrial barcodes [10,16,17]. Beyond that, other research teams were able to assemble mitochondrial genomes for butterflies [18] and nuclear genomes for flies and beetles [6], but both of these studies included material < 35 years old, which is not representative for most historical and type collections in museums. Representative studies using older insect museum specimens include, for example, the application of RAD tag and whole genome shotgun sequencing to a small sample of 50–85 year-old flies and ants [19], as well as a study by Suchan et al. [20] testing a newly developed RAD sequencing protocol (HyRAD—hybridization RAD) on butterfly and grasshopper specimens between 7–108 years old.

Neither single-locus barcoding nor whole-genome approaches are particularly suitable for arthropod systematics research—the former because it does not provide sufficient phylogenetic resolution and the latter because it is too costly. RAD tag sequencing is mainly useful to generate large amounts of SNP data for use in population level analyses. New phylogenomic methods that rely on target sequence capture and multiplexed next-generation sequencing to cost-effectively generate many hundreds of molecular markers are more appropriate for systematic studies, but have not yet been applied to arthropod museum specimens. A recently developed target sequence capture approach involving ultraconserved elements (UCEs) may be the best approach for arthropod museum specimens. Ultraconserved elements are highly conserved gene regions, scattered throughout the genomes of most organisms [21] and flanked by regions of greater sequence variability, which make them useful at different phylogenetic levels [22,23]. This technique has already been developed for use within hymenopteran insects [24], and it was successfully used to obtain UCE loci from museum bird specimens up to 120 years old [12]. We have used UCEs for phylogenomic systematics of ants on the generic [25] and species level [26], but our studies were restricted to specimens collected within the last 20 years.

Our goal for the present study was to test the efficacy of our UCE protocol on much older pinned insect museum specimens. We selected as our test case the large carpenter bees, genus *Xylocopa*, a group well represented in the National Museum of Natural History’s (NMNH) Entomology collection. Large carpenter bees are members of the family Apidae, with about 500 species distributed worldwide. These medium to large bees (ca. 12−30 mm) nest mostly in solid wood, into which they drill tunnels and line with cells for the development of their larvae [27]. Most species are solitary, but some species aggregate or even share nests [28]. *Xylocopa* are recognized as agriculturally important pollinators [29,30], but the evolutionary relationships among species remain poorly known, with only two studies available based on very limited taxon and gene sampling [31,32]. The diverse *Xylocopa* collection housed at NMNH offers the potential for a future large-scale, museum-based phylogenomic analysis of these bees, but material for many species is limited to very old specimens collected many decades ago. Here, we apply a target enrichment and sequence capture approach for UCEs to generate up to 972 loci for 51 pinned *Xylocopa* specimens of varying ages (≤121 years), in order to test the suitability of the method for a larger museum-based study. In most cases, we included both newer and older specimens representing the same species in order to confirm the integrity and robustness of UCE phylogenomic data generated from the older material. Given our previous observations indicating that genetic work on arthropod samples of age >20 years begins to be problematic, we divided our samples into old and more recently (<20 years) collected specimens, and compared pre- and post-library DNA concentration, and sequence and locus capture rates, and tested the correlation of these metrics with specimen age. In addition, we tested for the presence of a specific age or “breakpoint” in our data at which the effect of age on DNA quality might increase or decrease. Our inferred UCE phylogeny moreover offers first insights into species relationships and builds the foundation for a more extensive phylogenomic study on large carpenter bees.

## Materials and Methods

### Taxon Sampling and DNA Extractions

We extracted DNA from 72 pinned specimens from the National Museum of Natural History (NMNH) Entomology collection for this study. We plucked middle legs from the pinned bees using a pair of sterilized forceps and washed the tissue in 95% ethanol to remove dust, pollen, and other forms of accumulated debris on the bee legs. After evaporation of the ethanol (by drying the tissue on a clean Kimwipe^**™**^), the samples were placed in a freezer for several hours. DNA was then extracted destructively by grinding the frozen tissue with a sterile pestle, using a DNeasy Blood and TissueKit (Qiagen, Valencia, CA, USA) and following the manufacturer’s protocol, except the DNA was eluted in 130μL ddH_2_O instead of the supplied buffer. We ran 10μL of each extract for 60 min at 100 volt on 1.5% agarose SB (sodium borate) gels, to estimate size of the genomic DNA.

**Fig 1:**
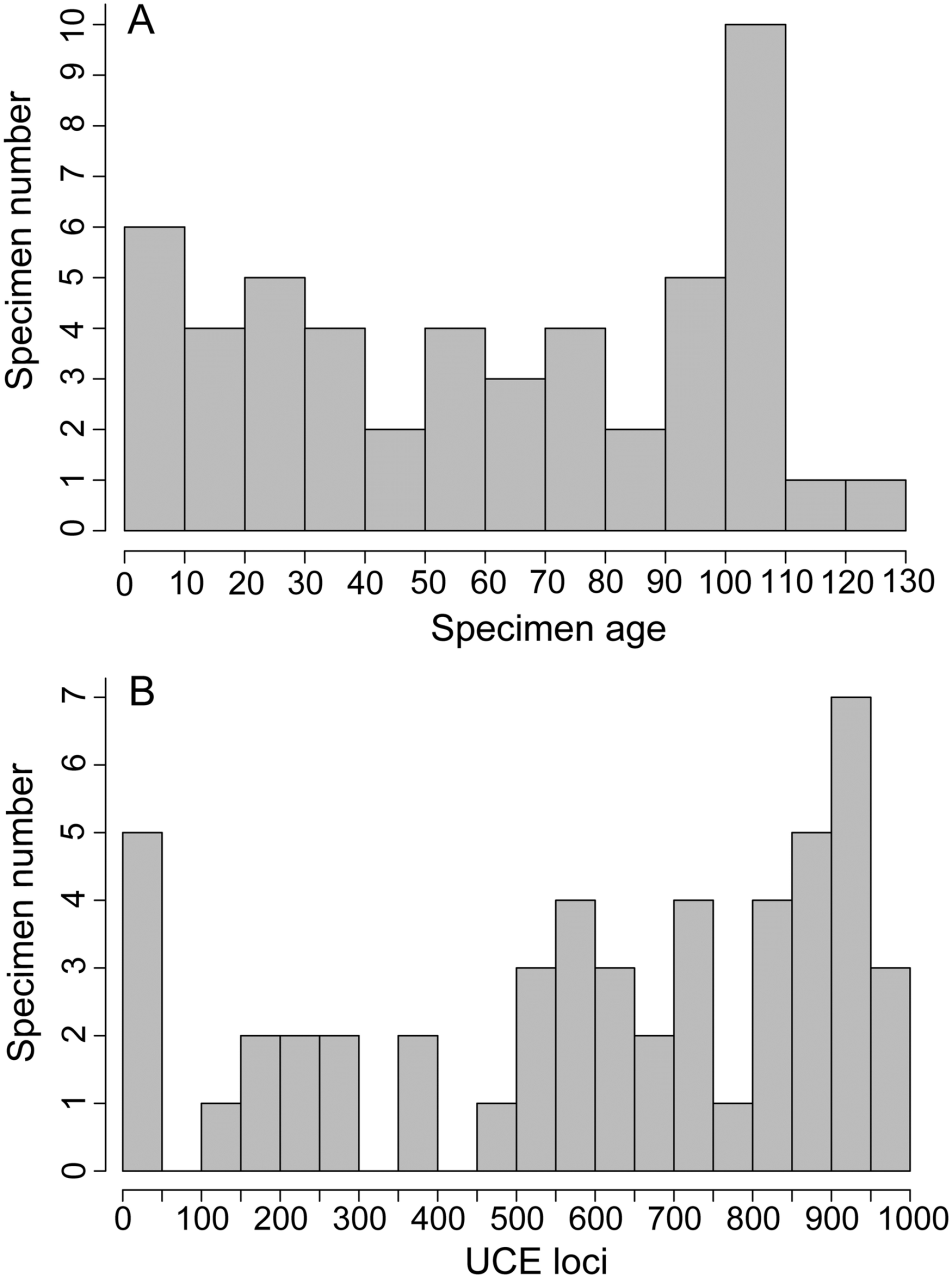
Histograms for specimen age and UCE locus count for the 51 taxa included in the study. **(A)** Specimen age distribution among sampled specimens; **(B)** Distribution of UCE loci capture.

From a pool of 60 successful extractions (12 extractions produced no quantifiable DNA), we chose to include 51 samples in this study, representing 25 *Xylocopa* species and nine subgenera, with collection dates ranging from 1894–2013 (aged 2–121 years, see Table 1 and Fig 1A). Twenty-two of these species are represented by two or more specimens. These conspecific individuals were selected by maximizing the age difference, i.e., we chose the oldest and the most recently collected specimen available for each species, although for several species no recent material (younger than 20 years) was available. Because of considerable size variation between species in the genus *Xylocopa*, we measured head width (HW) and mesotibial length (LMT) as an indicator of relative specimen size and tissue input (Table 1).

**Table 1:** Library preparation and UCE sequence capture statistics. Table listing for each taxon included in the study: the pre-library preparation DNA concentration and total DNA input values, the post-library preparation DNA concentration, the specimen age, the total raw read count, the total number of recovered contigs, their coverage (X) and mean length, and the number of assembled UCE contigs and their mean length and mean coverage. Individuals marked with * are represented in statistical analyses, but not in phylogenies. LMT = length of mesotibia, HW = head width. The UCE reads on target are the number of reads aligning to UCE loci/total reads.

								**All contigs**			**Contigs aligned to UCE loci**			
***Xylocopa* species**	**Age**	**LMT**	**HW**	**Pre-Lib. conc. (ng/μL)**	**DNA input (ng)**	**Post-Lib. conc. (ng/μL)**	**Total read count (bp)**	**Count**	**Avg. coverage (x)**	**Avg. length (bp)**	**Locus count**	**Avg. coverage (x)**	**Avg. length (bp)**	**Reads on target (%)**
*X*. *aestuans_25*	108	2.8	5.4	0.10	10.0	5.46	683,668	4283	25.4	286.7	202	21.4	260.2	0.052
*X*. *aestuans_26*	2	2.5	5.2	2.66	200	34.2	1,607,036	87552	12.7	328.3	890	74.9	880	22.6
*X*. *amethystina_28*	18	1.6	4.2	0.37	35.6	43.3	3,416,440	116185	14.4	322.6	915	143.1	604.7	20.8
*X*. *appendiculata_1*	94	2.8	5.0	0.23	23.3	2.88	70,256	1842	8.3	274.2	116	11.6	264.6	10.3
*X*. *appendiculata_49*	91	3.9	4.9	0.31	30.5	12.8	2,517,405	63392	14.5	308.2	972	136.8	466.4	28.3
*X*. *appendiculata_50*	46	2.8	5.1	0.27	26.7	7.04	1,557,369	27697	19.4	302.6	874	66.1	386.2	22.4
*X*. *aruana_51*	71	2.6	5.6	0.48	47.5	7.27	420,669	6306	11.2	544.2	10	11.9	261.4	0.1
*X*. *aruana_52*	33	2.7	5.5	0.46	45.7	8.03	332,369	1962	29.2	291.4	215	25.6	258.3	14.9
*X*. *caffra_30*	17	2.6	6.0	1.61	161	18.6	2,265,176	160226	9.1	363.3	814	134.6	938.6	22.1
*X*. *caffra_53*	106	3.2	6.3	0.24	23.7	5.71	1,806,465	26305	13.3	322.9	604	31.9	294.4	4.3
*X*. *calens_32*	25	2.5	4.7	0.49	49.3	14.9	564,738	17115	17.8	323.6	937	48.0	465.4	31.6
*X*. *calens_54*	103	2.4	5.1	2.20	200	5.04	485,665	5162	23.5	311	581	21.1	292.7	16.8
*X*. *californica_4*	17	2.7	5.7	1.47	147	24.8	2,396,700	183264	7.6	366.7	865	159.2	958	27
*X*. *californica_55*	103	3.0	4.7	0.06	5.8	1.53	2,126,600	21466	16.7	326.8	194	34.1	263.8	1.9
*X*. *cubaecola_5*	103	2.3	4.4	0.13	12.8	23.1	1,384,571	18817	18.4	490.9	800	50.3	357.7	12.5
*X*. *cubaecola_6*	4	1.9	3.9	1.10	110	21.8	617,309	19646	16.6	338.7	747	19.1	452.7	7.9
*X*. *darwini_34*	26	2.3	4.9	0.24	23.9	16.8	691,447	23396	12.5	324.6	909	36.5	457.4	22.8
*X*. *darwini_58*	92	2.8	5.2	0.21	21.3	7.44	637,438	10437	14.7	297.3	397	32.6	263.3	13.5
*X*. *dejeanii_59*	53	2.8	5.6	0.34	34.4	5.78	2,823,589	64383	14.1	311.3	958	142.4	456.1	28.5
*X*. *dejeanii_8*	21	3.0	5.5	0.48	48	8.62	1,333,156	11317	22.3	298.8	556	41.7	285.1	12.8
*X*. *frontalis_10*	19	4.2	7.4	0.76	76.2	15.1	263,448	5956	17.6	322.7	707	19.3	348.5	22.7
*X*. *frontalis_9*	78	4.2	7.2	0.07	6.6	20.3	1,233,601	11263	24.4	570	571	39.3	305.5	7.1
*X*. *grisescens_35*	61	4.2	7.0	0.46	45.8	8.91	526,617	10076	19	317.8	614	38.0	332.8	18.7
*X*. *grisescens_36*	36	3.7	6.8	2.08	200	15	1,791,986	73474	11.9	356	561	92.7	514.6	11.4
*X*. lucida_37 *	92	2.3	5.1	0.07	7.0	0.21	574,196	19882	6	281.2	6	8.4	238.3	0
*X*. *lucida_38*	36	1.9	4.7	0.24	23.6	14.7	599,343	3732	71.2	291.4	548	40.7	284.9	25.1
*X*. *micans_39*	101	2.3	n/a	0.35	34.8	8.41	538,417	10376	21.6	294.9	606	25.0	320.3	16.7
*X*. *micans_40*	24	2.1	4.8	0.41	41.0	10.6	3,479,137	178594	11.7	350.8	851	128.8	861.3	20.5
*X*. *mordax_11*	103	2.6	5.6	0.19	19.3	21	1,503,132	22099	15.4	333.3	687	25.5	387.4	6.8
*X*. *mordax_12*	2	3.1	6.4	9.80	200	37.5	2,458,642	161439	7.8	422	910	108.8	966.2	16.5
								**ALL Contigs**			**Contigs aligned to UCE loci**			
***Xylocopa* species**	**Age**	**LMT**	**HW**	**Pre-Lib. conc. (ng/μL)**	**DNA input (ng)**	**Post-Lib. conc. (ng/μL)**	**Total read count (bp)**	**Count**	**Avg. coverage (x)**	**Avg. length (bp)**	**Locus count**	**Avg. coverage (x)**	**Avg. length (bp)**	**Reads on target (%)**
*X*. *muscaria_60*	110	2.1	4.5	0.20	19.7	1.82	509,127	2392	27.2	287.9	37	33.7	255.1	2.5
*X*. *muscaria_61*	30	1.8	4.3	0.67	67.3	11	1,663,663	24426	31.9	300	923	100.7	422.3	36.7
*X*. *ruficornis_16*	43	3.6	6.1	0.87	86.8	22.9	350,971	2411	60.1	315.5	531	18.5	295.9	17.9
*X*. *sonorina_18*	3	2.9	5.2	2.17	200	29.3	1,127,835	59738	11.8	330.8	891	62.6	842.8	25.5
*X*. *sonorina_64*	104	3.0	6.0	0.07	6.5	1.94	1,458,862	4898	44.1	294	160	33.2	281.5	3.5
*X*. *tenuiscapa_41*	73	3.9	7.3	1.43	143	12.4	484,998	11890	16.2	292.6	452	31.1	311.4	14.3
*X*. *tenuiscapa_42*	38	4.4	6.3	5.19	200	22.1	819,943	23437	20	308.2	835	68.6	473.9	29
*X*. valga_19 *	114	3.8	5.4	0.07	6.8	4.77	431,707	1737	37.8	294.7	40	19.6	252.7	1.8
*X*. *valga_20*	8	3.0	5.4	7.37	200	59.1	1,242,975	80479	11.7	324.1	960	66.3	837.4	25.4
*X*. *valga_27*	121	2.9	5.1	0.15	14.5	21	1,545,331	70213	7	302.7	661	23.3	371.7	4.8
*X*. *varipuncta_43*	69	2.5	5.2	1.38	138	8.83	1,530,542	63806	11	324.1	730	43.8	468.9	10.7
*X*. *varipuncta_44*	29	3.0	5.3	0.46	45.6	11.9	1,947,619	64563	10	304.8	845	67.5	423.4	16.5
*X*. *violacea_67*	108	3.1	4.7	0.26	25.5	12.5	650,981	3019	59.7	312.4	366	35.6	266	11.4
*X*. *virginica_2*	13	2.5	5.6	3.55	200	28.7	3,354,921	248227	9	393.3	841	182.4	1041.6	21.6
*X*. *virginica_23*	90	2.6	5.4	0.19	18.9	5.45	215,120	1843	22.2	276.6	263	15.7	293.7	19.5
*X*. *virginica_24*	2	2.8	5.6	3.26	200	39.1	1,645,132	112814	6.9	365.7	909	90.0	909.4	22.4
*X*. *virginica_45*	53	2.5	5.8	0.38	38.0	7.65	951,600	29184	11.8	291.5	722	50.9	364.3	21.9
*X*. *viridigastra_69*	87	2.8	5.0	0.10	9.7	1.88	1,459,240	62843	6.2	322.5	43	7.4	269.5	0.1
*X*. *viridigastra_70*	55	2.9	4.9	0.63	63.1	1.81	737,049	3394	17.2	296.2	278	22.1	263.9	13.2
*X*. *viridis_71*	96	1.8	3.7	0.15	14.8	7.62	663,696	2442	56.1	312.5	528	43.3	273.6	34
*X*. *viridis_72*	51	1.9	3.7	0.06	5.8	9.73	1,714,798	38668	11.8	316.1	936	80.0	430.8	28.7
**Average Total**	**58**	**2.8**	**5.4**	**1.11**	**70.9**	**14.67**	**1,278,680**	**44707**	**20.0**	**330.2**	**599**	**56.2**	**446.6**	**16.2**
**Min**	**2**	**1.6**	**3.72**	**0.06**	**5.8**	**0.21**	**70,256**	**1737**	**6.0**	**274.2**	**6**	**7.4**	**238.3**	**0.0**
**Max**	**121**	**4.4**	**7.38**	**9.80**	**200.0**	**59.10**	**3,479,137**	**248227**	**71.2**	**570.0**	**972**	**182.4**	**1041.6**	**36.7**

The 51 voucher specimens for this study are housed at the NMNH in Washington, DC, with USNM specimen identifiers and collection data listed in S1 Table, and are publicly accessible in this repository. This study focuses on historical insect museum specimens collected throughout the last century, which were obtained following the necessary regulations for specimen collection, export, and import at the time of their accession into the NMNH collection. No new permits were required for the described study.

### Library Preparation, Target Enrichment and Sequencing of UCEs

We followed library preparation and target enrichment protocols by Faircloth et al. ([24], but see also [25]). DNA was quantified for each sample using a Qubit fluorometer (High sensitivity kit, Life Technologies, Inc.) and 5.8–200 ng DNA was sheared for 0–60 secs (amp = 25, pulse = 10) to a target size of approximately 500–600 bp by sonication (Q800 or Diagenode BioRuptor; Qsonica Inc.), depending on prior degradation and fragmentation of DNA. Forty-four highly degraded samples were sheared 10 secs or less. The sheared DNA was used as input for a modified genomic DNA library preparation protocol (Kapa Hyper Prep Library Kit, Kapa Biosystems), incorporating “with-bead” cleanup steps [33] and a generic SPRI substitute ([34], “speedbeads” hereafter), as described by Faircloth et al. [24]. For adapter ligation, we used TruSeq-style adapters [35] and PCR amplified 50% of the resulting library volume (15 μL) with a reaction mix of 25 μL HiFi HotStart polymerase (Kapa Biosystems), 2.5 μL each of Illumina TruSeq-style i5 and i7 primers (5 μM each), and 5 μL double-distilled water (ddH20). We used the following thermal protocol: 98°C for 45 s; 13 cycles of 98°C for 15 s, 65°C for 30 s, 72°C for 60 s, and final extension at 72°C for 5 m. After rehydrating (in 23 μL pH 8 Elution Buffer (EB hereafter) and purifying reactions using 1.0X speedbeads, 8–10 libraries were combined at equimolar ratios into enrichment pools with final concentrations of 107–190 ng/μL.

We enriched each pool using a set of 2749 custom-designed probes (MYcroarray, Inc.) targeting 1510 UCE loci in Hymenoptera (see Faircloth et al. [24]). We followed library enrichment procedures for the MYcroarray MYBaits kit [36], except we used a 0.1X concentration of the standard MYBaits concentration and added 0.7 μL of 500 μM custom blocking oligos designed against our custom sequence tags. We ran the hybridization reaction for 24 h at 65°C, subsequently bound all pools to streptavidin beads (MyOne C1; Life Technologies), and washed bound libraries according to a standard target enrichment protocol [36]. We used the with-bead approach for PCR recovery of enriched libraries, as described by Faircloth et al. [24]. We combined 15 μL of streptavidin bead-bound, enriched library with 25 μL HiFi HotStart Taq (Kapa Biosystems), 5 μL of Illumina TruSeq primer mix (5 μM forward and reverse primers) and 5 μL of ddH2O. We ran post-enrichment PCR using the following thermal profile: 98°C for 45 s; 18 cycles of 98°C for 15 s, 60°C for 30 s, 72°C for 60 s; and a final extension of 72°C for 5 m. We purified resulting reactions using 1.0X speedbeads, and we rehydrated the enriched pools in 22 μL EB. We quantified 2 μL of each enriched pool using a Qubit fluorometer (broad range kit).

Enrichment was verified by amplifying seven UCE loci (for primers see [24]) targeted by the probe set. We set up a relative qPCR by amplifying two replicates of 1 ng of enriched DNA from each library at all seven loci and comparing those results to two replicates of 1 ng unenriched DNA for each library at all seven loci. We performed qPCR using a SYBR^®^ FAST qPCR kit (Kapa Biosystems) on a ViiA^™^ 7 (Life Technologies). Following data collection, we computed the average of the replicate crossing point (Cp) values for each library at each amplicon, and we computed fold-enrichment values, assuming an efficiency of 1.78 and using the formula 1.78abs (enriched Cp—unenriched Cp). We then created serial dilutions of each pool (1:200,000, 1:800,000, 1:1.000,000, 1:10.000,000) and performed qPCR library quantification, assuming an average library fragment length of 600 bp. Based on the size-adjusted concentrations estimated by qPCR, we pooled libraries at equimolar concentrations and size-selected for 250–800 with a BluePippin (SageScience) where necessary. All of the UCE laboratory work was conducted in and with support of staff at the Laboratories of Analytical Biology (L.A.B.) facilities of the National Museum of Natural History. We sequenced the pooled libraries by performing two paired-end runs on an Illumina MiSeq housed at the L.A.B. Quality-trimmed sequence reads generated as part of this study are available from the NCBI Sequence Read Archive under SRA accession SRP072230 (http://www.ncbi.nlm.nih.gov/sra/SRP072230).

### Processing and Alignment of UCE Data

We removed adapter contamination and low-quality bases from the demultiplexed FASTQ data output using Illumiprocessor [37], based on the package Trimmomatic [38]. Our data processing relied on scripts from the PHYLUCE package [39,40]. We computed summary statistics for the data using the *get_fastq_stats*.*py* script and assembled the cleaned reads using the *assemblo_trinity*.*py* wrapper around the program Trinity ([41], version trinityrnaseq_r20140717). Average sequencing coverage across assembled contigs was calculated using *get_trinity_coverage*.*py*.

Species-specific contig assemblies were aligned to a FASTA file of all enrichment baits by the script *match_contigs_to_probes*.*py*. (min_coverage = 50, min_identity = 80), identifying assembled contigs representing enriched UCE loci from each species. We calculated sequence coverage statistics (avg, min, max) for contigs containing UCE loci with *get_trinity_coverage_for_uce_loci*.*py*. We used the script *get_match_counts*.*py* to query the relational database containing matched probes created in the previous step to generate a list of UCE loci shared across all taxa. We then used this list of UCE loci within the *get_fastas_from_match_counts*.*py* script to create FASTA files for each UCE locus, which contained sequence data for taxa present at that particular locus. We aligned all data in all these FASTA files using MAFFT [42] through *seqcap_align_2*.*py* (min-length = 20, no-trim). We trimmed this alignment with a wrapper script (*get_gblocks_trimmed_alignment_from _untrimmed*.*py*) for Gblocks [43] using the following settings: b1 = 0.5, b2 = 0.5, b3 = 12, b4 = 7. We selected two subsets of UCE alignments with the script *get_only_loci_with_min_taxa*.*py* with 50% and 70% completeness for taxa per locus. We added missing data designators to each file with *add_missing_data_designators*.*py*, and generated alignment statistics across all alignments using *get_align_summary_data*.*py*. For each subset, we concatenated individual alignments of UCE loci into one nexus alignment file with *format_nexus_files_for_raxml*.*py* for subsequent phylogenetic analyses. We calculated the number of gaps and missing data with the program AMAS [44].

### Statistical Analyses

We investigated the correlation between specimen age, library preparation statistics (pre-library DNA concentration, post-library DNA concentration), and sequence capture statistics (raw read count, UCE reads on target, UCE mean contig length and UCE locus count) by calculating the Pearson’s correlation coefficient between each of these variables. Similarly, we analyzed the relationship of body size (LMT and HW) with DNA extraction yield (i.e., pre-library DNA concentration) and UCE locus capture to investigate whether these variables were influenced by tissue input size rather than specimen age. To test preconceptions on specimen age and DNA quality and yield, we divided our 51 samples into two age groups (10 samples below 20 years = younger, 41 samples above 20 years = older) and compared means of the different variables with a Welsh two sample t-test. All analyses were performed with the *stats* package in R v3.2.1 [45]. To assess whether we could detect similar “breakpoints” in the relationship between specimen age and our DNA yield and sequence capture variables, we then performed piecewise (or segmented) regression with the R-package *segmented* [46]. We used specimen age as explanatory variable (*seg*.*Z*) and did not specify any breakpoints (by omitting *psi*).

### Phylogenetic Inference

We estimated a phylogeny for our *Xylocopa* data, primarily to assess the quality of the generated UCE sequences, but also to gain initial insights into relationships between *Xylocopa* subgenera and species. First, we selected data partitions using a development version of PartitionFinder [47] that depends on the software fast_TIGER (http://dx.doi.org/10.5281/zenodo.12914) and is designed to handle large genome-scale datasets. We then performed maximum likelihood best tree and bootstrap searches (N = 100) in RAxML v8.0.3 [48] on matrices that were 50% and 70% complete with regard to taxon representation for each locus (see above). We ran unrooted analyses on our *Xylocopa* dataset as well as rooted analyses that included previously published data from two apid taxa (*Bombus pennsylvanicus* and *Apis mellifera*; [24]). After preliminary analyses with all 51 taxa, we excluded two taxa (*X*. *lucida*_37 and X. *valga_19*) from final analyses because their phylogenetic position could not be estimated due to very low UCE locus capture. All of the above phylogenetic analyses were performed on the Smithsonian Institution high performance cluster (SI/HPC). The data matrices and resulting tree files are deposited in Treebase under accession number TB2:S19070 (http://purl.org/phylo/treebase/phylows/study/TB2:S19070).

## Results

### Extractions and UCE Capture

From the initial pool of 72 extractions, 60 samples yielded sufficient DNA (>0.05 ng/μL) to proceed with library preparation, and only 12 extractions produced no quantifiable DNA (with specimen ages ranging from 19−115 years). Based on gel electrophoresis, most of the older samples appeared to have very fragmented DNA, mainly 50−300bp. However, gel images were difficult to interpret conclusively in many instances because of low DNA concentrations. We prepared DNA libraries from 53 of the successful extractions. However, we later excluded two samples from data analyses due to possible species misidentifications. Pre- and post-library preparation values and UCE sequence capture statistics for the 51 bee samples included in the study are summarized in Table 1. Body sizes of bees ranged from 3.7–7.4 mm head width (HW) and 1.6–4.4 mm length of mesotibia (LMT). DNA sample concentrations ranged from 0.06–9.8 ng/μL pre-library preparation and from 0.21–59.1 ng/μL post-library preparation.

Multiplexed sequencing of UCEs captured 70,256–3,479,137 reads per sample. Trinity assembled 1,737–248,227 contigs per sample. These contigs had average lengths of 274.2–570 bp and sequencing coverage was 6–71.2X. From the total assembled contigs, we recovered 6–972 UCE loci per sample (Fig 1B gives an overview of the distribution) with average lengths per sample ranging from 238.3–1041.6 bp. The average coverage across the captured UCE loci per sample ranged from 7.4–182.4X.

### Correlation of UCE Capture with Specimen Age and DNA Quality

**Fig 2:**
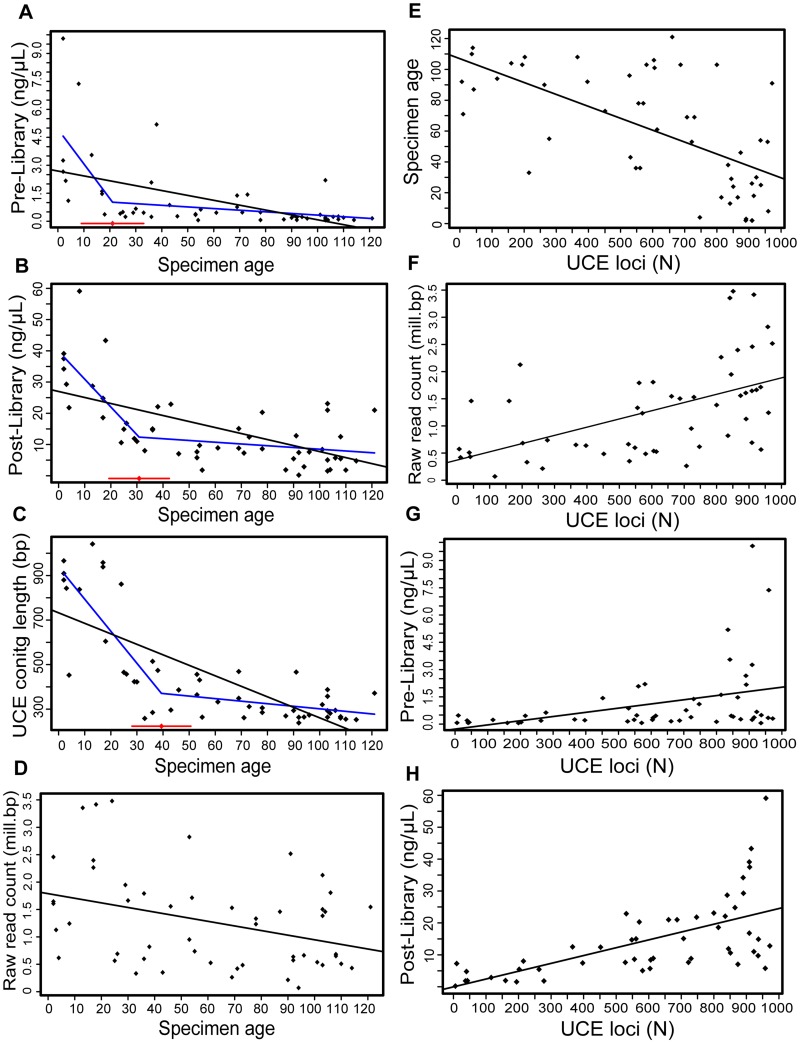
Correlation between specimen age, library preparation and sequence capture statistics. **(A)** pre-library DNA concentration (ng/μL) with specimen age (years); (**B)** post-library DNA concentration (ng/μL) with specimen age (years); (**C)** UCE contig length (bp) with specimen age (years); (**D)** raw read count (million bp) with specimen age (years); (**E)** specimen age (years) with UCE locus count (n loci); (**F)** raw read count (million bp) with UCE locus count (n loci); (**G)** pre-library DNA concentration (ng/μL) with UCE locus count (n loci); (**H)** post-library DNA concentration (ng/μL) with UCE locus count (n loci). Correlations were tested with the Pearson’s correlation coefficient; black lines represent linear regressions; see Table 2 for specification of results. Blue lines in panels A, B and C represent piecewise regression lines, and red line with diamond represents the estimated breakpoint with 95% confidence interval around it (Table 3).

**Fig 3:**
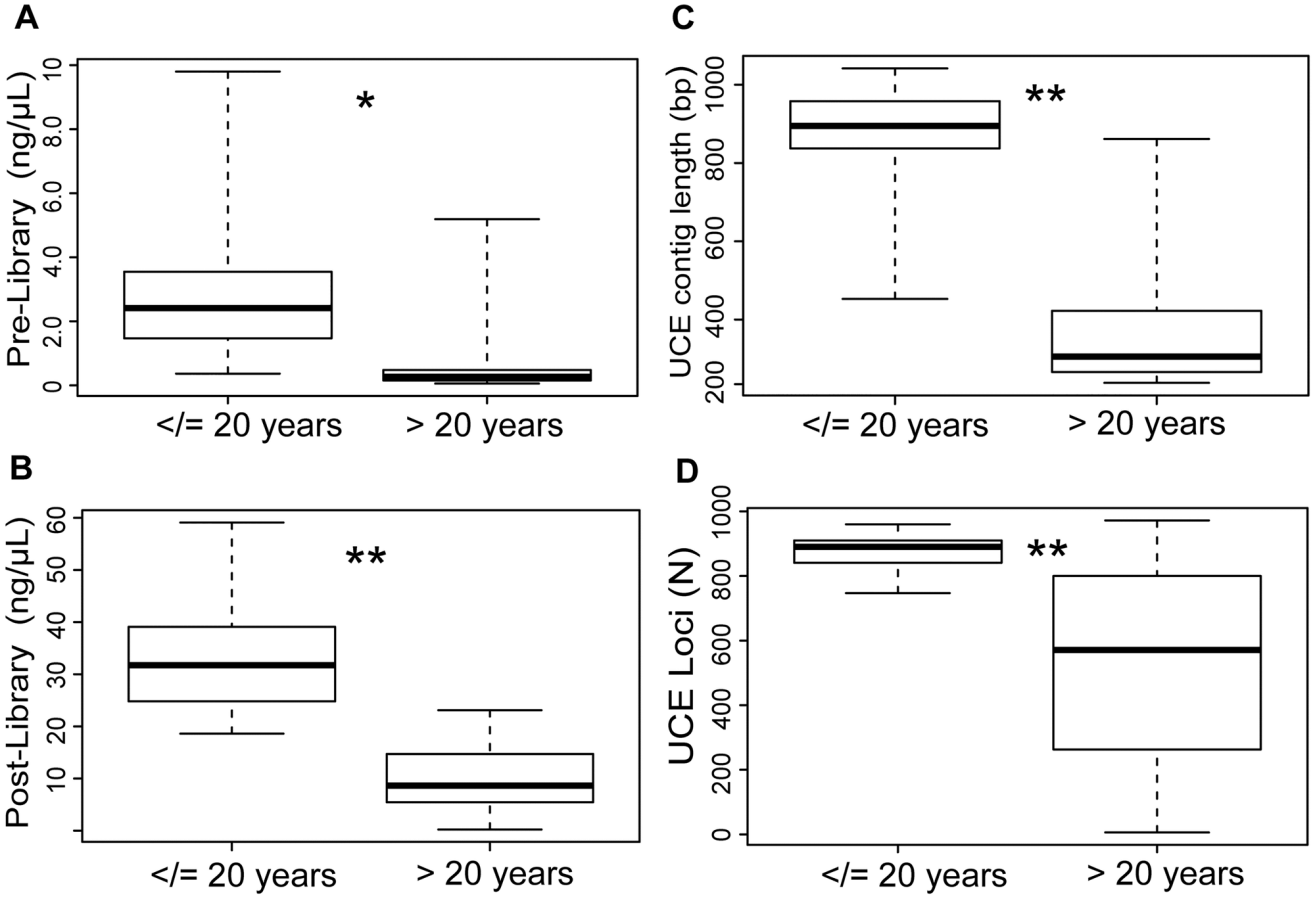
Boxplots comparing means of several library preparation and UCE capture statistics by age group. We compared means of two age groups, above 20 years (N = 41) and below 20 years (N = 10), with a Welsh two sample t-test for (**A)** pre-library DNA concentration (ng/μL), (**B)** post-library DNA concentration (ng/μL), (**C)** UCE contig length (bp), and (**D**) UCE locus count (n loci). ** = p<0.001, * = p<0.05; see Table 3 for full results of t-tests.

We found that all investigated variables (pre-library DNA concentration, post-library DNA concentration, raw read count, UCE locus count, UCE mean contig length and UCE reads on target) decreased with increasing specimen age, and this negative correlation was highly significant in all cases (R = -0.36–0.75 and p < 0.001; Table 2, Fig 2A–2E). Furthermore, the number of captured UCE loci was significantly positively correlated with all variables (R = 0.38–0.75 and mostly p <0.001; pre-library DNA concentration p-value <0.05; Table 2), and correlation strength increased with increasing pre-library DNA concentration (Fig 2G), post-library DNA concentration (Fig 2H), raw read count (Fig 2F), and UCE reads on target (not shown). Pre-library concentration was also significantly correlated with an increase in post-library concentration (R = 0.68; p <0.001) and increase in UCE contig length (R = 0.64; p <0.001), but it showed no relationship with the number of raw sequence reads recovered or the percentage of UCE reads on target (Table 2). We found no significant correlation between the tissue input quantity (as measured by our body size indicators LMT and HW) and DNA yield (as measured by pre-library concentration) and UCE locus capture. Comparisons of means for age groups showed significantly higher pre-library DNA concentration (t = 2.83; p = 0.016), post-library DNA concentration (t = 6.05; p<0.001), UCE contig length (t = 8.26; p<0.001) and locus counts (t = 6.13; p<0.001) for specimens of age 20 and less (see Table 3 and Fig 3). However, for pre-library DNA concentration and UCE contig length, both age groups show wide ranges of values (Fig 3A and 3C). By contrast, UCE locus capture showed less variation for younger than for older specimens (Fig 3D). Mean post-library DNA concentration was higher for younger specimens but with a wider range than for older specimens (Fig 3B). Piecewise regression supported breakpoints in our data between a 20–40 year range. For pre-library DNA concentration we recovered a breakpoint of 21 years (Table 3, Fig 2A). For post-library DNA concentration we recovered an estimate of 31 years (Table 3, Fig 2B), whereas for UCE contig length the breakpoint was 39 years (Table 3, Fig 2C). For UCE locus capture our piecewise regression analyses did not support a distinct breakpoint.

**Table 2:** Correlation between selected UCE capture statistics. Correlation between specimen age, DNA concentration (pre and post library preparation), raw sequence read counts, reads on UCE targets, UCE mean contig length, UCE locus count, as well body size (LMT and HW). Calculated as Pearson's product-moment correlation. ** = p <0.001, * p <0.05. See also Fig 2.

	LMT	HW	Pre-library concentr.	Post-library concentr.	Raw read count	UCE reads on target	UCE contig length	UCE locus count
**Specimen age**	/	/	-0.53**	-0.64**	-0.36**	-0.58**	-0.75**	-0.63**
**Pre-library DNA concentration**	-0.09	-0.03	/	0.68**	0.21	0.25	0.64**	0.38*
**Post-library DNA concentration**	/	/	/	/	0.35*	0.36**	0.72**	0.62**
**Raw read count**	/	/	/	/	/	/	0.64**	0.53**
**UCE reads on target**	/	/	/	/	/	/	0.44*	0.75**
**UCE contig length**	/	/	/	/	/	/	/	0.65**
**UCE locus count**	-0.09	-0.03	/	/	/	/	/	/

**Table 3:** Comparison of means by age group. Table comparing group means for specimens aged under 20 years and over 20 years for pre-PCR library concentration (ng/μL), post-PCR library concentration (ng/μL), UCE mean contig length (bp) and UCE locus count (n). Significance was tested with a Welsh two sample t-test. See also Fig 3.

Specimen age	≤20 years	>20 years	p-value	Breakpoint	St.Err
**Pre-library concentration**	3.10	0.56	0.017	21.04	5.96
**Post-library concentration**	33.64	10.05	1.07E-04	30.84	5.74
**UCE contig length**	843.1	349.9	5.29E-06	39.38	5.59
**UCE loci**	859	528	1.47E-07	/	/

### Phylogenetic Results

**Fig 4:**
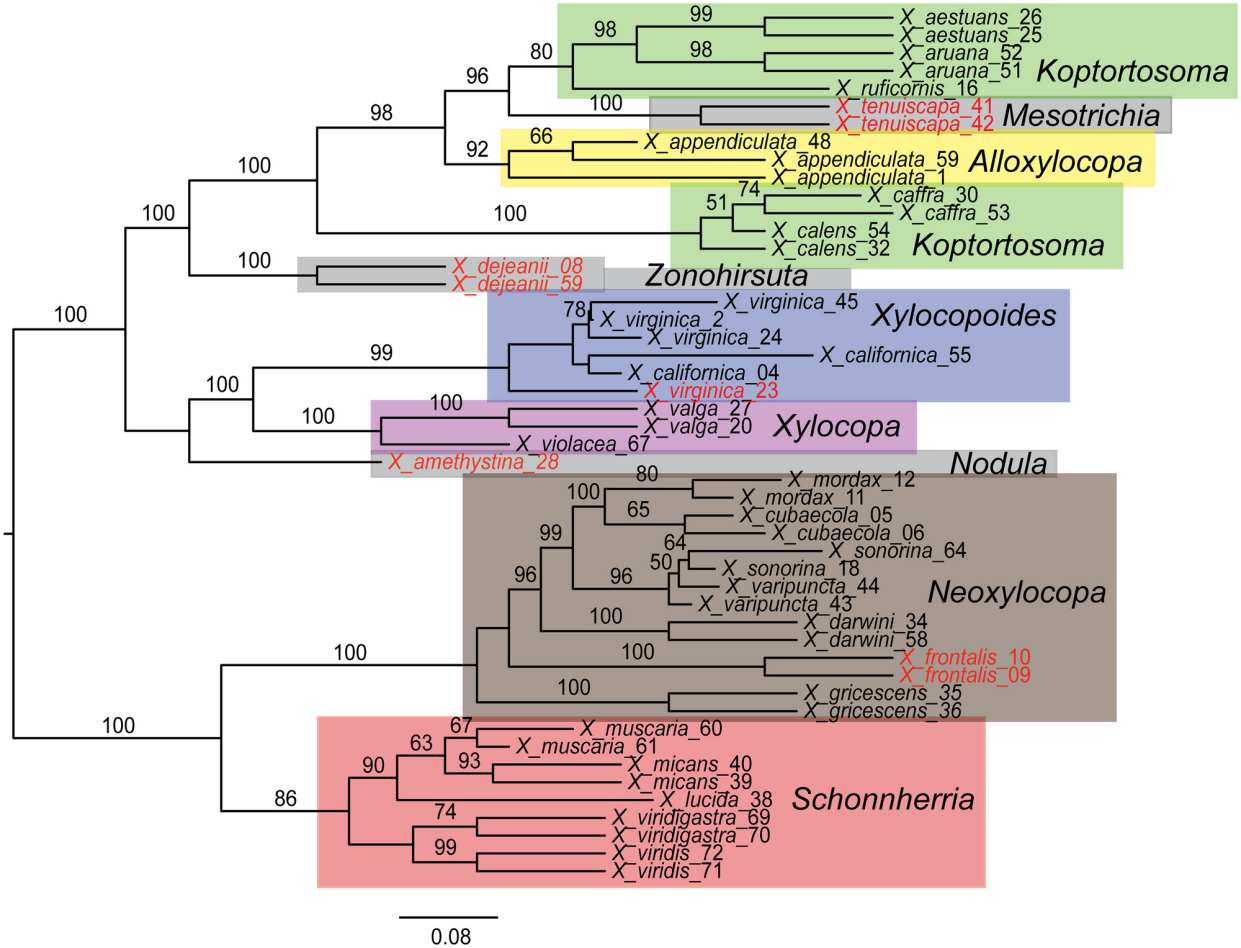
Phylogeny of *Xylocopa* study specimen based on the 70% complete data set. Maximum likelihood best tree including 49 *Xylocopa* specimens, based on 96 UCE loci and 34,309 bp (70% matrix). Values from bootstrap analysis are mapped onto the tree. Only bootstrap values > 50 are shown. Taxon labels in red font indicate taxa with a different position in the analysis of the 50% complete data set (Fig 5). The respective subgenera have been mapped on the phylogeny. Scale bars represent nucleotide substitutions per base pair; tree is unrooted and displayed using midpoint rooting.

**Fig 5:**
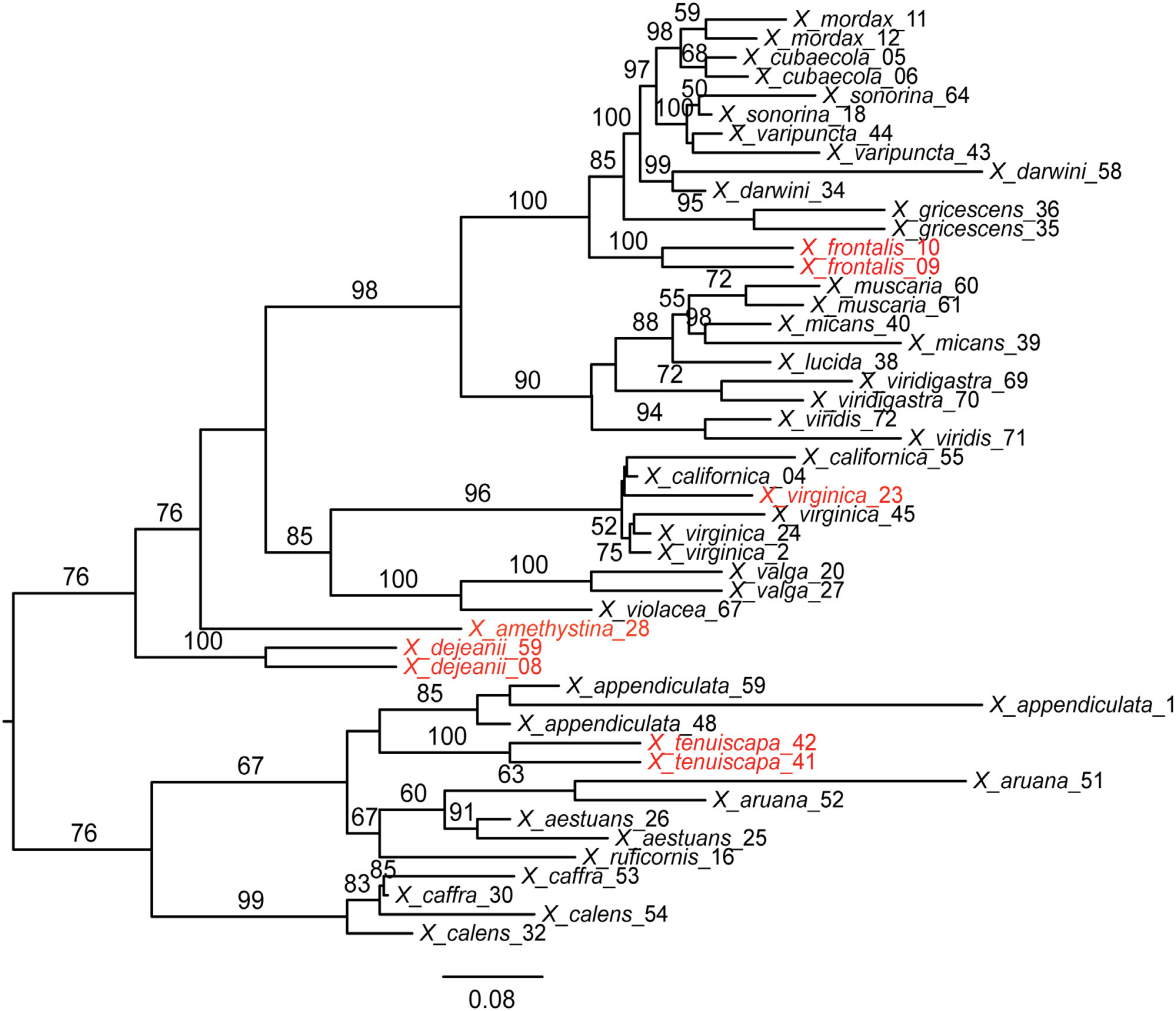
Phylogeny of *Xylocopa* study specimen based on the 50% complete data set. Maximum likelihood best tree including 49 *Xylocopa* specimens, based on 774 UCE loci and 258,013 bp (50% matrix). Values from bootstrap analysis are mapped onto the tree. Only bootstrap values > 50 are shown. Taxon labels in red font indicate taxa with a different position in the analysis of the 70% complete data set (Fig 4). Scale bars represent nucleotide substitutions per base pair; tree is unrooted and displayed using midpoint rooting.

Concatenation of the 50% complete matrix retained 774 loci for the 49 *Xylocopa* taxa (excluding outgroups and *X*. *lucida*_37 and X. *valga_19*), with an alignment length of 258,013 bp (45.6% missing data), whereas the 70% complete matrix contained 96 loci and a total length of 34,309 bp (33.5% missing data). PartitionFinder divided these matrices into 8 (50% matrix) and 5 (70% matrix) subsets. Figs 4 and 5 show maximum likelihood phylogenies estimated from these partitioned data sets; phylogenies rooted with the two other apid outgroups can be found in S1 Fig. Our conspecific samples grouped together in almost all cases, with the exceptions being *Xylocopa virginica*_23 and *X*. *calens*. For both of these exceptions, a second species previously considered to be closely related rendered these taxa paraphyletic, but without strong bootstrap support. Furthermore, species in the same subgenera formed clades in our phylogenies (Figs 4 and 5) except for species currently placed in *Koptortosoma*, which are separated into two distinct clades with strong support. Six taxa (highlighted taxon labels in Figs 4 and 5) change position between the 70% complete matrix (Fig 4) and the 50% complete matrix (Fig 5) analyses, but mostly these involve relationships that have received low support in either of the analyses. The most important difference between the two phylogenies involves the position of the *Xylocopoides* + *Xylocopa* clade. This clade is highly supported in the smaller, 94 loci data set as being the sister clade to *Koptortosoma*, *Zonohirsuta*, *Alloxylocopa* and *Mesotrichia*. In the 50% complete analysis this clade groups with *Neoxylocopa* and *Schonnherria*, albeit with very low support. Overall, the 70% matrix generated a much better resolved and supported phylogeny (Fig 4), and deeper relationships seem to degrade in the 50% phylogeny (Fig 5). For example, the positions of *X*. *tenuiscapa* and *X*. *dejeanii* are both highly supported in the 70% analysis, but receive low support for altered relationships in the 50% analysis. Several long branches are introduced in the 50% tree, for example in *X*. *appendiculata*_1 (Fig 5). The analyses including the apid outgroup taxa return similar results regarding shallow species-level relationships. However, overall these analyses recovered very poor resolution in the backbone of the *Xylocopa* phylogeny (S1 Fig).

## Discussion

Ultraconserved elements (UCEs) have become increasingly popular markers in phylogenomics, and have been used to generate genomic-scale datasets from the skins of museum bird specimens [12]. Targeted capture methods such as the UCE approach can generate phylogenomic data from older museum collections more cheaply and efficiently compared to whole genome or transcriptome approaches [49]. Within the arthropods, target UCEs have been developed for Hymenoptera [24], but this technique has so far been only applied to recently collected material that was mostly stored in ethanol under climate-controlled conditions [25,26]. Given the reported success on bird museum specimens, we investigated how this method would perform on small, dried insect specimens, and tested this in the present study using a data set of large carpenter bee specimens. We were able to successfully generate UCE sequences from specimens ranging up to 121 years old. Specimen age had a negative effect on the DNA quality measures and sequence capture statistics we investigated; however, we found a surprising amount of variation in this relationship and an indication for breakpoints after which DNA degradation may proceed at slower speeds. We here discuss these results regarding the performance and promise of museum insect specimens in reconstructing phylogenies.

### Age-Related Factors of UCE Sequence Capture

Our extractions of genomic DNA from old museum specimens had a 83% success rate (48 out of 58 extractions of specimens >20 years produced quantifiable DNA), while the success rate for more recent material was only marginally higher with 86% (12 of 14 extractions of specimens ≤20 years). The UCE method was effective in capturing up to 972 loci from these dried and pinned bees of up to 121 years age. Unsurprisingly, specimen age and metrics of DNA quantity (pre-library DNA concentration, post-library DNA concentration) and sequence capture rates (raw read count, UCE locus count, UCE mean contig length and UCE reads on target) were negatively correlated. Metrics of DNA concentration were correlated with overall sequencing success, which confirms results from previous studies [11,50]. Pre- and post-library DNA concentrations were a better indicator for UCE contig length than for total UCE locus capture. Moreover, specimen age had a stronger adverse effect on UCE contig length than on total UCE locus capture, confirming findings of McCormack et al. [12] for UCE capture from museum bird specimens. Remarkably, we were able to generate ~1.5 million reads and 661 UCE loci for the oldest specimen in our data set (*X*. *valga*_27: 121 years; Table 1), and recovered the highest locus count (972) from a specimen with age of 91 years (*X*. *appendiculata*_49; Table 1). However, both of these taxa had relatively low average UCE contig lengths (372 bp and 466 bp, respectively); therefore, less data was recovered for those samples.

Recently collected material (≤20 years) generally showed better performance with regard to pre-library DNA concentration, post-library DNA concentration, UCE mean contig length, and UCE locus capture rates than older material (>20 years; Table 3 and Fig 3). A study on spiders across a time series of museum samples (up to 63 years old) also reported 20 years to be the specimen age after which sequencing success decreased [16]. However, this study focused only on PCR amplification of *cytochrome oxidase I*, and samples used in this study had been stored in ethanol, whereas ours were dried. Our piecewise regression analysis sheds additional light on the relationship of specimen age and the quantitative DNA measures. We found breakpoints in our data, after which the negative correlation of age and the DNA metrics drastically decreased (Fig 2A–2C). This result has interesting implications because it points to a rapid degradation of DNA quality until about 20−40 years after collection and mounting of the specimens. Beyond this limit, further aging (at least of our dried samples) had limited effect on the quantitative measures of DNA yield and UCE contig length. However, it should be noted that we were not able to identify a breakpoint in the relationship of age and UCE locus capture. In that case, the negative correlation was maintained across the entire data set, which indicates that other variables also may have had an important effect. For example, our input DNA may have been too fragmented even for this next-generation sequencing approach (also suggested by decreasing contig lengths), in which case consequently fewer loci will be recovered and assembled. Our protocol was optimized for sheared DNA of sizes 300–500 bp and many of our samples were likely below this size range, despite the minimal sonication, if any, applied to these degraded samples. A more accurate estimation of size ranges of input DNA in our study was unfortunately impeded by low DNA concentrations, which makes it difficult to visualize DNA with gel eletrophoresis. More sophisticated methods, such as running the samples on a TapeStation prior to library preparation could be used to measure DNA fragmentation, but these methods are generally too expensive to be used on a large scale.

The tremendous amount of variation in our results across old specimens was our most surprising result. Irrespective of specimen size, pre-library concentrations ranged from 0.06−2.2 ng/μL, and locus count from 6−972 loci. An influential factor here, besides age, may be the individual history of the specimens. The specimens presently reside in a climate-controlled collection at NMNH, but it is quite likely that historically specimens were exposed to a less favorable climate that may have exacerbated degradation in some samples more than others. Moreover, we have little to no information how the specimens were killed and collected. Carpenter bees are large and conspicuous and thus usually hand collected (with a net), but less favorable killing agents such as ethyl acetate vapor and/or preservatives such as propanol or methanol, which lead to increased degradation of DNA [51,52], could have been used prior to specimen pinning. All of our pinned samples were desiccated, but the speed at which the desiccation took place could have considerable impact on DNA preservation. Rapidly dried specimens have been found to preserve DNA longer [15], but it is unclear over what timescales this factor matters.

### Using Museum Specimens to Reconstruct Phylogenies

A major concern when extracting and sequencing DNA from museum specimens is the possibility of contaminating the sample with DNA from other organisms [53]. We tested the reliability of our UCE data by including multiple representatives from 22 *Xylocopa* species and reconstructing a phylogeny for our samples. Twenty of the twenty-two conspecific specimen pairs or triplets in our phylogenetic analysis grouped together, and the two that did not both formed poorly supported paraphyletic groups relative to a second species thought to be closely related (Fig 4). We thus conclude that no contamination problems exist in our data. Our success in generating a robust phylogeny from *Xylocopa* museum specimens is also supported by the result that the *Xylocopa* subgenera here are all recovered as monophyletic with the exception of *Koptortosoma*, for which polyphyly was already indicated with strong Bayesian support by Leys and Hogendoorn [54]. The overall subgeneric relationships recovered in our phylogeny also broadly agree with the results recovered in these previous studies.

Another important consideration when working with sequence data from old museum specimens is the amount of missing data introduced by variability in sequencing success. We analyzed our UCE data by filtering for presence of taxa per loci and thus generated and analyzed two data sets of very different size and level of completeness (70% and 50% complete matrix). Our 70% complete matrix retained only 94 UCE loci, a relatively small number because our data set showed large variation in UCE locus count. In contrast, a published UCE data set limited to recently-collected ant samples with the same filters applied retained 951 loci [25]. Our 50% matrix retained a level of magnitude more data, 774 loci, but we found this data set produced a phylogeny with reduced support (Fig 5) and introduced changes in tree topology between the two analyses (highlighted taxa in Figs 4 & 5). We suggest that the higher percentage of missing data (45.6% versus 33.5%) in the 50% matrix is responsible for the differing results and favor the results of the more complete 70% matrix. Two specimens with very low locus counts (*X*. *lucida_37*: 6 loci and *X*. *valga_19*: 40 loci) had to be excluded from all our analyses because they were not successfully placed in the phylogeny. In contrast, all taxa in our data set with locus counts above 40 were firmly placed in the phylogeny. A certain threshold value of above 40–50 loci recovered per taxon could therefore be useful to decide whether to include or exclude the taxon in question from phylogenetic analyses. However, three other taxa with equally low sequence capture success (*X*. *aruana_51*, *X*. *muscaria_60*, *X*. *viridigastra_69* with 10, 37, and 43 loci, respectively; Table 1 and Figs 4 & 5) were firmly placed with their conspecific counterparts in our analyses. Thus even taxa with lower amounts of data could be correctly anchored in the phylogeny, presumably as long as the loci that are captured are shared with closely related species. We pose this question for further investigation—aided by future comparative data from other systems—whether to exclude taxa with fewer loci from phylogenetic analyses based on a predetermined threshold criterion, or to include even these very low yield samples in order to increase taxon sampling and thereby gain additional phylogenetic insights, while risking in turn a potentially disruptive behavior of these taxa in the analyses.

## Conclusions

We were able to generate nearly 1000 UCE loci from pinned bee specimens ranging from ages up to 121 years old. While all our investigated variables of DNA quality and sequence capture declined with increasing specimen age, this negative correlation was not as clear-cut and strong as expected. We found breakpoints in the relationship of specimen age and DNA quality and length of recovered contigs between 20−40 years after specimen collection, indicating that during this time DNA degradation is most prominent and progressing faster. Adequate, conditioned specimen storage during this age may increase the “shelf-life” of pinned insect specimens for longer time periods. All DNA quality variables measured showed moderate correlation with locus capture and were more strongly correlated with UCE contig length. Despite the drawback of capturing shorter contigs from older specimens, our protocol still generates large amounts of data from samples with very low starting DNA concentrations and presumably high degradation and fragmentation.

Furthermore, we found our UCE approach highly successful for the reconstruction of phylogenies for insect museum specimens—out of 51 sequenced samples, only two with very low counts of captured UCE loci failed to give reasonable phylogenetic results—demonstrating that this method is well suited for larger scale investigations of museum insect phylogenomics. We did extract DNA from relatively large insects, where one leg yields more tissue than is available from crushing the entire body of most ants, for example. Thus, it remains now to be tested whether sufficient input DNA can also be obtained from smaller dried insect specimens. Nonetheless, our results are encouraging and suggest that UCE phylogenomics could revolutionize molecular arthropod systematics through the large-scale utilization of historical museum specimens.

## Supporting Information

S1 Fig Additional trees from analyses including outgroups. **(A)** Maximum likelihood best tree of 51 *Xylocopa* specimens, based on 828 UCE loci and 268566 bp (50% matrix), with values from bootstrap analysis mapped onto this tree. **(B)** Maximum Likelihood best tree of 51 *Xylocopa* specimens, based on 123 UCE loci and 42753 bp (70% matrix), with values from bootstrap analysis mapped onto this tree. Only bootstrap values > 50 are shown. Scale bars represent nucleotide substitutions per base pair; trees are rooted with *Apis mellifera* and *Bombus pennsylvanicus*.
(TIF)

S1 Table Collection data of *Xylocopa* specimens included in the study. Summary of collection information for the 51 *Xylocopa* specimens sequenced in this study, including USNMENT voucher number, collection date, country, locality and coordinates. All information has been transcribed from label data and amended for clarity. Wherever possible, localities have been georeferenced.
(PDF)
