# Supplementary figures and images for: Sequence Capture and Phylogenetic Utility of Genomic Ultraconserved Elements Obtained from Pinned Insect Specimens
Source: PLoS One. 2016 Aug 24;11(8):e0161531. doi: 10.1371/journal.pone.0161531 (PMC4996520; doi:10.1371/journal.pone.0161531)

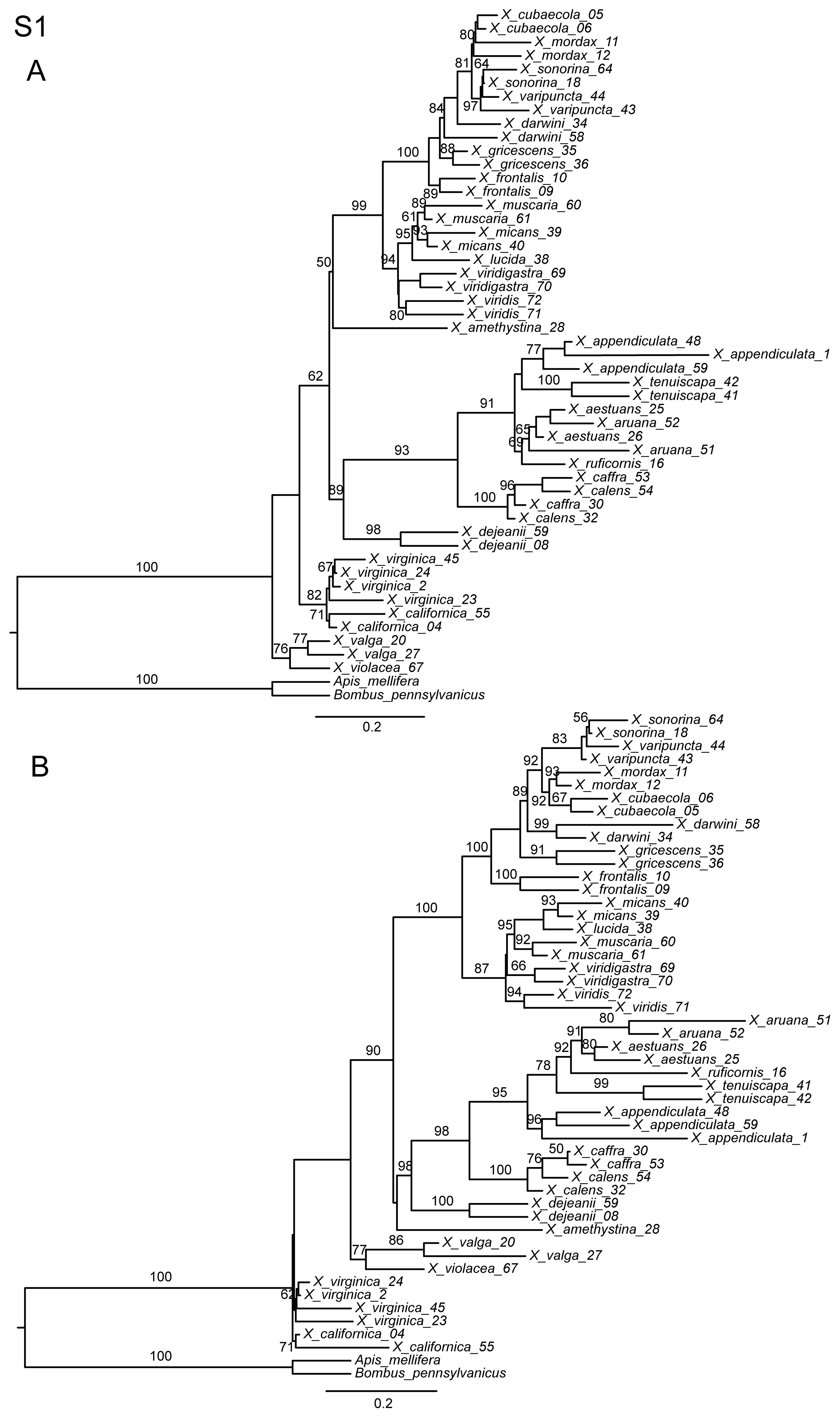

Supplement: S1 Fig — (A) Maximum likelihood best tree of 51 Xylocopa specimens, based on 828 UCE loci and 268566 bp (50% matrix), with values from bootstrap analysis mapped onto this tree. (B) Maximum Likelihood best tree of 51 Xylocopa specimens, based on 123 UCE loci and 42753 bp (70% matrix), with values from bootstrap analysis mapped onto this tree. Only bootstrap values > 50 are shown. Scale bars represent nucleotide substitutions per base pair; trees are rooted with Apis mellifera and Bombus pennsylvanicus. (TIF) [file pone.0161531.s001.tif]
