# Supplementary material for: Sequence Capture and Phylogenetic Utility of Genomic Ultraconserved Elements Obtained from Pinned Insect Specimens
Source: PLoS One. 2016 Aug 24;11(8):e0161531. doi: 10.1371/journal.pone.0161531 (PMC4996520; doi:10.1371/journal.pone.0161531)
Supplement: S1 Table — Summary of collection information for the 51 Xylocopa specimens sequenced in this study, including USNMENT voucher number, collection date, country, locality and coordinates. All information has been transcribed from label data and amended for clarity. Wherever possible, localities have been georeferenced. (PDF) [file pone.0161531.s002.pdf]

S1 Table: Collection data of *Xylocopa* specimens included in the study. Summary of collection information for the 51 *Xylocopa* specimens sequenced in this study, including USNMENT voucher number, collection date, country, locality and coordinates. All information has been transcribed from label data and amended for clarity. Wherever possible, localities have been georeferenced.

USNMENT	*Xylocopa* species	Collection date	Collector	Country	Locality	Latitude	Longitude
1124004	*aestuans_25*	1906-1907	T. Barbour	Indonesia	Java: Bogor [Buitenzorg]	-6.60000	106.80000
1124005	*aestuans_26*	2013	unknown	n/a	n/a	n/a	n/a
1124013	*amethystina_28*	25-26/iv/1997	A.W. & J.W. Norden	Sri Lanka	Badulla Dist.: Mahiyangana	7.24930	81.00011
1124018	*appendiculata_1*	06/v/1921	A.P. Jakot	China	Peking [?]	n/a	n/a
1161817	*appendiculata_49*	ix/1924	D.C. Graham	China	Sichuan: Yibin [Suifu]	28.76667	104.61667
1161818	*appendiculata_50*	29/iv/1969	K. Tsuneki	Japan	Fukui[?]	n/a	n/a
1124022	*aruana_51*	1944	K.V. Krombein	New Guinea	Milne Bay: KB Mission	n/a	n/a
1124023	*aruana_52*	17/v/1982	Darlington (Harvard Exp.)	Australia	Queensland: Cape York	n/a	n/a
1124045	*caffra_30*	19/i/1998	E. Grissell	South Africa	Western Cape Prov.: Cederberg Wilderness Area (19km E Clan Williams)	n/a	n/a
1161819	*caffra_53*	2/vi/1909	unknown	South Africa	Western Cape Prov.: Cape Town: Lion's Head	-33.93472	18.38917
1124047	*calens_32*	1/-2/iv/1990	W.E. Steiner	Madagascar	Antananarivo, Manakambahiny	n/a	n/a
1161810	*calens_54*	xii/1912	Lang & Chapin	Congo	Faradje	3.73503	29.70997
1124049	*californica_4*	16/ix/1998	M. & P.H. Arnaud	USA	AZ: Cochise Co, Chiricahua Mts., Rustler Pk	31.90450	-109.27950
1161811	*californica_55*	1912	G. Randall	USA	CA: Contra Costa Co.	n/a	n/a
1124062	*cubaecola_5*	1912	G.Link	Cuba	Isla de la Juventud [Isle of Pines]: Nueva Gerona	n/a	n/a
1124063	*cubaecola_6*	9/vi/2011	S.W. Droege	Cuba	Guantanamo Bay	19.93481	-75.11390
1124069	*darwini_34*	22-28/iii/1989	Peck & Sinclair	Ecuador	Galapagos: Floreana, 5km E Black Beach, 250m	-1.29750	-90.43417
1161812	*darwini_58*	1/iv/1923	unknown	Ecuador	Galapagos: Santa Cruz Isl. [Indefatigable]	-0.63333	-90.36667
1161813	*dejeanii_59*	28/iv/1962	E.S. Rosss & D. Cavagnaro	Malaysia	Kedah	n/a	n/a
1124071	*dejeanii_8*	1937	Mann (NGS SI expedition)	Indonesia	Sumatra: Dolok Silau	3.07000	98.72000
1124077	*frontalis_10*	14/iii/1946	H.H. Stage	Suriname	Marowijne Dist.: Moengo	5.61667	-54.40000
1124076	*frontalis_9*	24/x/1937	L. J. Stannard	Panama	Canal Zone: Albrook Field	8.97590	-79.55553
1124080	*grisescens_35*	18/viii/1954	A.G.A. Silva	Brazil	Rio Grande do Norte	n/a	n/a
1124081	*grisescens_36*	17/vi/1979	A. Raw	Brazil	Tocatins: Bananal Isl.	-11.33333	-50.68333
1124106	*lucida_37*	xii/1923	H. Bassler	Peru	Middle Rio Ucayali	-9.99900	-74.08900
1124107	*lucida_38*	2-5/xi/1979	J.B. Heppner	Peru	Madre de Dios, Rio Tambopata Reserve	-12.92056	-69.28194
1124112	*micans_39*	23/iv/1914	D. Kaki	USA	FL: Glen St. Mary	30.27611	-82.16139
1124113	*micans_40*	21/x/1991	R.J. McGinley & M.J. Mello	USA	AL: Baldwin Co., Ft. Morgan	30.24990	-87.69994
1124114	*morda11*	24/ii/1912	C. W. Hooker	USA	PR: Mayaguez	18.20111	-67.13972
1124115	*morda12*	24/iv/2013	S. Prado	USA	PR: St. Isabel	17.99101	-66.44338
1161814	*muscaria_60*	7/ii/1905	unknown	Guatemala	Los Amates	15.26667	-89.10000
1161805	*muscaria_61*	3-10/v/1985	Menke & Carpenter	Venezuela	Guarico: Hato Masaguaral	8.66989	-67.57085
1124133	*ruficornis_16*	8-10/iii/1972	K.v. Krombein & P.B. Karunaratne	Sri Lanka	Hambantota Dist.: Palatupana	n/a	n/a

1124147	*sonorina_18*	3-4/iii/2012	S.W. Droege	USA	Hawaii: Oahu	21.32633	-157.68434
1161806	*sonorina_64*	28/ix/1911	Hy.H. Severin	USA	Hawaii: Oahu	n/a	n/a
1124158	*tenuiscapa_41*	1942	W.L. Jellison	China	Yun Hsien [?]	n/a	n/a
1124159	*tenuiscapa_42*	9/xii/1977	D. Pletsch	Indonesia	Jambi Prov.: Sungai Bengkal	n/a	n/a
1124164	*valga_19*	v/1901	P. Wechsler	Algeria	Blida, Atlas Mts.	36.47528	2.82833
1124165	*valga_20*	1/vii/2007	M.G. Rightmyer, H. Oezbek	Turkey	Erzurum Prov.: 5 km NE Pasinier	n/a	n/a
1124012	*valga_27*	1894	E. Turkestan	China	Xinjiang Prov.: Yarkand	n/a	n/a
1124170	*varipuncta_43*	9/viii/1946	P.D. Hurd	USA	CA: Riverside Co.: Blythe	33.61028	-114.59639
1124171	*varipuncta_44*	24/vii/1986	F.A. Ehrenford	USA	CA: Kern Co.: Kern Park	n/a	n/a
1124172	*violacea_67*	iii/1907	Friese	Italy	South Tyrol: Bozen	n/a	n/a
1124019	*virginica_2*	11/5/02	D&W.N. Mathis	USA	VA: Chesterfield, Pocahontas State Park	37.37885	-77.56767
1124178	*virginica_23*	21/05/1925	J.B. Cronin	USA	NJ: Chatsworth	39.81750	-74.53500
1124179	*virginica_24*	5/6/13	USGS-DRG	USA	MD: Prince George's Co.	38.97240	-76.87433
1124176	*virginica_45*	10/vii/1962	J.F. Reinert	USA	OK: Garfield County	n/a	n/a
1161808	*viridigastra_69*	15/iii/1928	R.C. Shannon	Peru	Lima, Verrugas Canyon	n/a	n/a
1161809	*viridigastra_70*	iii/1960	J. Förster	Ecuador	Quito	-0.23333	-78.51667
1124182	*viridis_71*	11/xi/1919	unknown	Paraguay	Alto Parana: Puerto Bertoni	n/a	n/a
1124183	*viridis_72*	7/xi/1961	F.M. Oliviera	Brazil	Real Forte do Príncipe da Beira	n/a	n/a
